# Functional, Biological and Nutritional Properties of Protein Fraction Isolated from *Yarrowia lipolytica* Biomass

**DOI:** 10.3390/foods14213801

**Published:** 2025-11-06

**Authors:** Marek Szołtysik, Anna Mandecka, Marcelina Maciejewska, Anna Dąbrowska, Marek Nowak

**Affiliations:** Department of Development Functional Food Products, Faculty of Biotechnology and Food Science, Wroclaw University of Environmental and Life Sciences, 50-366 Wroclaw, Poland; marek.szoltysik@upwr.edu.pl (M.S.); marcelina.maciejewska@upwr.edu.pl (M.M.); anna.dabrowska@upwr.edu.pl (A.D.); marek.nowak1@upwr.edu.pl (M.N.)

**Keywords:** *Yarrowia lipolytica* yeast, enzyme hydrolysis, bioactive peptides, functional properties

## Abstract

This study evaluated the nutritional, functional, biological, and sensory potential of proteins derived from *Yarrowia lipolytica* biomass and their enzymatic hydrolysates for food applications. Three strains were cultivated under bioreactor conditions, with strain JII1c selected for its superior biomass yield and protein content. Its amino acid composition was rich in lysine and branched-chain amino acids, with protein quality indices (CS = 37.8%, EAAI = 36.17%) confirming value in plant-based diets. Proteins were isolated and hydrolysed using a non-commercial serine protease from *Cucurbita ficifolia*, which enhanced solubility (NSI: 19.4 → 49.2%), water and oil absorption, and emulsion stability. Hydrolysates showed notable biological activities, including ACE (71.8%), DPP-IV (52.3%), and α-glucosidase (67.4%) inhibition, indicating potential metabolic benefits. Sensory evaluation of extrudates confirmed improvements in aroma, texture, and flavour when hydrolysates were incorporated. The use of a plant-derived protease demonstrates a sustainable approach to producing bioactive peptides. *Y. lipolytica* hydrolysates emerge as promising clean-label ingredients that combine nutritional quality with techno-functional performance, supporting their integration into health-oriented and sustainable food products.

## 1. Introduction

The global demand for high-quality protein is rising due to protein–energy malnutrition, amino acid imbalance, climate change, and shifting dietary preferences. Conventional protein sources such as meat, dairy, and fish, though rich in essential amino acids, contribute to environmental degradation through greenhouse gas emissions, land use, and water consumption [1,2]. Despite overall growth in food output, many populations still lack access to adequate diets, reflecting deficiencies of food systems that prioritize yield and cost-efficiency over nutritional quality [3]. Meanwhile, vegetarian, vegan and flexitarian trends are driving demand for novel protein ingredients that are both sustainable and nutritionally balanced. In the United States, plant-based foods reached 61% household penetration in 2021, with 19% buying meat substitutes [2,4,5,6,7].

Alternative proteins from plants, insects, microalgae and fungi are gaining attention for their environmental and functional potential [8,9]. Among microbial sources, *Yarrowia lipolytica* is particularly promising, offering up to 60% protein per dry weight when cultivated under optimised bioreactor conditions using agro-industrial by-products [10]. Its proteins exhibit a complete amino acid profile, with high levels of lysine and leucine, surpassing even egg protein in lysine content [5,11]. *Y. lipolytica* has been recognized as safe for food and feed applications, holding GRAS status in the United States and approval as a novel food by the European Food Safety Authority (EFSA, 2019), confirming its safety, metabolic versatility, and high productivity [12]. Additionally, its cell wall provides bioactive polysaccharides and B vitamins such as riboflavin and B12, aligning with clean-label and sustainability trends [5,13,14].

This yeast also supports circular economy strategies by valorising waste streams, including bread, brewer’s spent grain and lignocellulosic residues, without extensive pretreatment [15,16,17]. Such processes reduce environmental burden while producing protein-rich biomass for food and feed applications.

Protein hydrolysis is an effective approach to enhance the biofunctionality of yeast proteins, releasing bioactive peptides (BAPs) with antioxidant, antihypertensive, antimicrobial and anti-inflammatory effects [18]. Functional properties of hydrolysates depend on substrate composition and processing conditions [19]. Plant-derived enzymes are particularly attractive for sustainable applications. A serine protease from *Cucurbita ficifolia* (Asian pumpkin) has been shown to generate peptides with strong antioxidant and functional properties and has been successfully applied to substrates including casein and insect protein [20,21,22,23]. Recent studies confirm that *Y. lipolytica* hydrolysates obtained via enzymatic treatment display potent antioxidant activity and functionality as flavour enhancers, emulsifiers and foaming agents. For example, Gottardi et al. (2022) demonstrated that hydrolysates obtained with *Y. lipolytica* strain YL2 showed strong DPPH radical scavenging activity, reaching 86.4% after 72 h of incubation, confirming the high bioactive potential of peptides derived from this yeast [5,14,24,25].

The aim of this study was to evaluate the potential of *Y. lipolytica* proteins and their enzymatic hydrolysates in food applications. The research focused on optimising cultivation and biomass processing for high protein yield, followed by enzymatic hydrolysis to generate bioactive peptide fractions. The nutritional, functional and sensory properties of both protein isolates and hydrolysates were assessed to explore their value in plant-based formulations.

## 2. Materials and Methods

### 2.1. Substrates

The material used for the study was the biomass of *Yarrowia lipolytica* yeast, which was obtained from bioreactor cultures (*Yarrowia lipolytica* yeast was obtained from the cultures collection of the Department of Biotechnology and Food Microbiology, Wroclaw University of Environmental and Life Sciences). The protein was then isolated from this biomass. Three distinct yeast strains were utilised for the culture of *Y. lipolitica*: JII1c, JII1a, and PII6a. The medium used for the reactor cultures was of plant origin, i.e., it was a vegan product.

### 2.2. Bioreactor Culture

Cultures were grown in a BioFlow 310 bioreactor (New Brunswick Scientific GmbH, Nurtingen, Germany) with a total volume of 7 L and a working volume of 4 L at a temperature of 28 °C. Aeration was maintained at 1 vvm, and agitation was set at 500 rpm for 48–72 h. The culture medium consisted of (g/L): yeast extract (1.7), casein (4.0), refined fatty acids (10.0), KH_2_PO_4_ (0.5), MgSO_4_·7H_2_O (0.25) and NH_4_Cl (1.0). Inoculum propagation was conducted sequentially in three bioreactors of increasing volume. In the first stage, a 7 L BioFlow 310 bioreactor (working volume: 4 L) was operated under the same temperature, aeration (1 vvm), and agitation (500 rpm) conditions for 48 h. The second stage involved a 50 L MPPF bioreactor (New Brunswick Scientific Co., Inc., Edison, NJ, USA) with a working volume of 4 L, maintained at identical operating conditions. In the third stage, cultivation was continued in the same 50 L bioreactor with an increased working volume, while aeration was reduced to 0.6 vvm and agitation adjusted to 350 rpm. The resulting concentrate was subjected to spray-drying with the addition of 3% (*w*/*w*) methylcellulose as a carrier. Spray-drying was performed at an inlet air temperature of 140 °C and an outlet air temperature of 88 °C, with a feed flow rate of 2.8 L/h and an air pressure of 1.1 bar. Biomass was harvested based on optical density. Optical density was reported based on the standard curve, for which extinction was measured for a specific amount expressed as [log10 CFU/mL]. Three replicates were performed for each strain.

### 2.3. Nutritional Quality Assessment of Biomasses

The obtained biomasses were analysed for their content of dry matter, protein, carbohydrates (including dietary fibre and sugars), fat and fatty acids, and both macro- and microelements [26,27,28,29,30,31,32]. B vitamins were determined by HPLC after enzymatic digestion and saponification, using a C18 column with UV/fluorescence detection [33,34,35,36,37,38]. Determination of the content of sodium was carried out in an acetylene/air flame by atomic emission spectrometry using the SpectraAA atomic absorption spectrometer with the flame attachment AA240FS (Varian). The methods were validated using certified reference material; the measurement uncertainty was estimated at 5%. Mineralization was performed in accordance with the Polish Standard PN-EN 13805:2003 “Foodstuffs. Determination of trace elements. Pressure mineralization” [39].

The amino acid content was analysed by reversed-phase high-performance liquid chromatography (RP-HPLC). Samples (500 μL) were solubilised in Tris/HCl buffer with urea and 2-mercaptoethanol, loaded on a Zorbax XDB-C18 column (250 × 4.5 mm, Agilent Technologies, Inc., Santa Clara, CA, USA), and separated at 1 mL/min, 30 °C, with gradient elution (0–100% acetonitrile/TFA); detection at 230 nm [40].

To assess the nutritional quality of the yeast biomasses, the method described by Liang et al. was applied [41]. Essential amino acid (EAA) profiles were analysed and compared to a reference protein (whole egg). Two parameters were calculated: the Chemical Score (CS) and the Essential Amino Acid Index (EAAI).

The CS was determined as the ratio of each essential amino acid present in the protein sample to its corresponding content in whole egg protein, multiplied by 100. The final CS was expressed as the geometric mean of these individual ratios, according to the equation:

(1) $$Chemical\;score=\left(\frac{EAA\;in\;sample}{EAA\;in\;whole\;egg}\right)x100$$

The essential amino acid index (EAAI) reflects the overall balance of essential amino acids relative to the reference protein and provides a holistic metric for protein quality. The reference amino acid pattern of whole egg protein was adopted from FAO/WHO/UNU (1985) [42]. The EAAI was calculated as the geometric mean of the ratios of each essential amino acid in the sample to its corresponding content in whole egg protein. The calculation followed the formula:

(2) $$EAAI=100x\sqrt[{n}]{\frac{{Lys}_{p}}{{Lys}_{s}}x\frac{{Trp}_{p}}{{Trp}_{s}}x\ldots x\frac{{Val}_{p}}{{Val}_{s}}}$$

where

p—refers to the protein sample;

s—refers to the standard (whole egg protein);

n—is the number of essential amino acids considered (with Met + Cys and Phe + Tyr treated as combined pairs).

The energy value of biomass was calculated using the Atwater general factors (4 kcal/g for protein, 9 kcal/g for fat, and 4 kcal/g for carbohydrates), based on the measured contents of macronutrients, according to FAO/WHO recommendations and Codex Alimentarius guidelines [43].

### 2.4. Microbiological Analysis

The microbiological safety of the dried *Yarrowia lipolytica* biomass was evaluated according to international standards. Total aerobic mesophilic bacteria (TAMC) were determined using ISO 4833-1:2013 [44]. Total yeast and mould count (TYMC) was measured according to ISO 21527-2:2008 [45]. The presence of viable *Yarrowia lipolytica* cells was confirmed using YPD agar (yeast extract–peptone–dextrose) incubated at 28 °C for 48 h, with colony morphology verified microscopically and through Gram staining. Coliform bacteria were detected based on ISO 4832:2006 [46]. *Salmonella* spp. detection followed ISO 6579-1:2017 [47]. Results were expressed as presence or absence in 25 g of sample.

### 2.5. Protein Extraction from JII1c Biomass

The protein isolates (PI) were obtained from *Yarrowia lipolytica* biomass JII1c using a two-step procedure. Initially, the yeast biomass, suspended in water, was subjected to acid hydrolysis to disrupt the cell walls and release intracellular components. In the first step, NaOH was used at a concentration of 1 M (t = 60 min), and in the second step, the 1 M HCl was used (t = 15 min). Following cell disruption, proteins were extracted under alkaline conditions (pH = 12) to increase their solubility. Subsequently, protein precipitation was induced by adjusting the pH to the isoelectric point (pH = 4.5), as described by Shetty K. J. & Kinsella (1979) [48]. During both steps, mechanical stirring was used. The protein content was determined post-precipitation by the Kjeldahl method. The efficiency of the entire process was estimated at approximately 55–60%.

### 2.6. Enzymatic Hydrolysis (EH)

#### 2.6.1. Enzyme Applied in the Study

Serine proteinase was isolated from Asian pumpkin (*Curcubita ficifolia*) by the method according to Dryjanski et al., 1990 [49] Proteolytic activity was determined using 2% casein in Tris/HCl (pH 8.6), incubated for 10 min at 35.5 °C. The reaction was stopped with 5% TCA, centrifuged at 5500× *g* (RCF) for 10 min, and absorbance was measured at λ = 280 nm. One unit was defined as a 0.1 increase in absorbance.

#### 2.6.2. Hydrolysis Procedure

Protein isolates (PI) from *Yarrowia lipolytica* JII1c biomass were subjected to enzymatic hydrolysis (EH) using pepsin 107,192 (Sigma-Aldrich, Poznań, Poland) and and non-commercial serine protease extracted from *Cucurbita ficifolia.* Enzymes were added at 150 enzyme units (U) per mg of protein to 1% substrate protein solution.

Hydrolysis was conducted at 37 °C, and samples as protein hydrolysates (PHs) were collected at 0, 5, 12, and 24 h for further analyses. The enzyme was thermally inactivated at 100 °C. After inactivation, the hydrolysates were centrifuged (5000× *g*; 20 min) and then frozen. The hydrolysates were captured at −18 °C and resuspended at room temperature before analysis.

The control consisted of protein isolates (PIs) not treated with enzymes but subjected to the same thermal conditions and incubation times as the enzymatic samples. The hydrolysis conditions were established based on previously published studies describing the catalytic characteristics *of Cucurbita ficifolia* serine protease [20,21,22,23].

#### 2.6.3. Degree of Hydrolysis (DH) [%]

The degree of hydrolysis (DH) was determined according to the method of Silvestre (1996), based on the concentration of peptides soluble in 5% trichloroacetic acid (TCA) [50]. Briefly, 1 mL of hydrolysate was mixed with 1 mL of 10% TCA and allowed to stand for 1 h to precipitate unhydrolyzed proteins. The samples were then centrifuged at 5000× *g* for 15 min at 5 °C. Protein concentration in the obtained supernatant was measured spectrophotometrically at wavelengths of λ = 280 and λ = 235 nm. The degree of hydrolysis was expressed as the relative increase in soluble peptide content after enzymatic treatment compared to the control sample.

### 2.7. Determination of Antioxidant Activity

#### 2.7.1. DPPH

Antioxidant activity was determined by the modified Yen & Chen (1995) method by adding 1 mL of 96% ethanol and 500 μL of a 0.3 mM ethanolic DPPH (2,2-di(4-tertoctylphenyl)-1-picrylhydrazyl) radical solution to 1 mL of peptide solution in Tris-HCl buffer at pH 7.0, with mixing [51]. Absorbance was measured at λ = 517 nm after 30 min of incubation. The antioxidant activity of a 1 mg/mL peptide solution was determined on the basis of a standard curve prepared for Trolox and expressed as [μM Trolox/mg].

#### 2.7.2. Ferric Reducing Antioxidant Power Assay (FRAP)

Antioxidant activity was determined as the ability of the hydrolysate to reduce the Fe(III) to Fe(II) ions in reaction with TPTZ (2,4,6-Tris(2-pyridyl)-s-triazine) [52]. To 1 mL of an aqueous peptide solution was added 3 mL of a working solution containing 1A:1B:10C, where A (10 mM TPTZ in 40 mM HCl), B (20 mM FeCl_3_ × 6 H_2_O), C (0.3 M acetate buffer pH 3.6 (3.1 g C_2_H_3_NaO_2_ × 3H_2_O, 16 mL acetic acid/1 L H_2_O). The samples were incubated for 10 min at room temperature, and then the absorption was measured at λ = 593 nm. Antioxidant activity was expressed as the ability to reduce the oxidation state of iron ions—Fe(III) to Fe(II). The concentration of Fe(II) ions in the sample was calculated based on a standard curve prepared for specific concentrations of FeSO_4_ solution. The reducing capacity of enzymatic hydrolysates and peptides was calculated per 1 mg of protein. The results were expressed as [g Fe^3+^/mg].

#### 2.7.3. Fe(II) Ion Chelation

Chelation of iron ions was determined by colorimetric measurement of Fe(II) not bound by hydrolysate in a reaction mixture using ferrozine (3-(2-pyridyl)-5,6-diphenyl-1,2,4-triazine-p,p′-disulfonic acid monosodium salt hydrate) [53]. To 250 μL of appropriately diluted peptide solution was added 1250 μL of H_2_O and 110 μL of 1 mM FeCl_2_ (9.94 mg FeCl_2_ in 50 mL H_2_O). After 2 min of incubation, 1 mL of 0.5 mM ferrozine solution (12.31 mg ferrozine in 50 mL H_2_O) was added. The samples were kept for 10 min at room temperature, after which absorbance was measured at λ = 562 nm. Simultaneously, a blank sample was performed, in which 1000 μL of distilled H_2_O and a reagent sample (110 μL of 1 mM FeCl_2_, 1190 μL H_2_O, 1000 μL H_2_O) were added. Iron ion chelating activity was expressed as micrograms of bound FeCl_2_ per milligram of protein [μg Fe^2+^/mg], based on a standard curve prepared with FeCl_2_ as standard.

#### 2.7.4. ACE Inhibitory Activity

The ACE inhibitory activity was determined according to the method of Hernández-Ledesma et al., with modifications made for the purpose of the study [54]. Briefly, 20 µL of each sample was added to 0.1 mL of 0.1 M potassium phosphate buffer (pH 8.3) containing 0.3 M NaCl and 5 mM Hip-His-Leu (HHL) as the substrate. After the addition of 5 milliunits (mU) of ACE (Angiotensin-Converting Enzyme, Sigma Aldrich, >2.0 U/mg protein), the reaction mixture was incubated at 37 °C for 30 min. The reaction was stopped by adding 0.1 mL of 1 M HCl. The hippuric acid formed was extracted with ethyl acetate, evaporated by heating at 95 °C for 10 min, redissolved in distilled water, and measured spectrophotometrically at λ = 228 nm. All assays were performed in triplicate. The inhibitory activity was expressed as the percentage of ACE inhibition at a given peptide nitrogen concentration, and the IC_50_ value (the concentration of peptides required to inhibit 50% of ACE activity) was calculated.

#### 2.7.5. α-Glucosidase Inhibitory Activity

According to Yu et al., peptides were incubated with α-glucosidase and substrate (pNPG) [55]. To 610 µL of 0.1 M potassium phosphate buffer, pH 6.8, were added 10 µL of 3 mM reduced glutathione solution, 5 µL of α-glucosidase (10 U/mL), and 10 µL of peptide solution. The mixture was preincubated for 20 min at 37 °C, after which the reaction was initiated by adding 10 µL of the substrate p-NPG (p-nitrophenyl β-D-glucopyranoside) (10 mM). The reaction was continued for 30 min at 37 °C. After this time, 650 µL of 1 M Na_2_CO_3_ was added to terminate the process. The amount of released product, p-nitrophenol, was determined spectrophotometrically at λ = 410 nm. The enzyme inhibitory activity (IC_50_) was expressed as the amount of substance required to half-inhibit (IC_50_) the activity of α-glucosidase under the conditions described above.

#### 2.7.6. DPP-IV Inhibitory Activity

Activity was determined according to Giovanni Tulipano’s method [56]. The test material was suspended in 0.1 M Tris-HCl buffer, pH 8.0. The sample (25 µL) was preincubated with the same volume of Gly-Pro-p-nitroanilide substrate (1.6 mM) at 37 °C for 10 min. Then, 50 µL of DPP-IV (0.01 U/mL, in 0.1 M Tris-HCl buffer, pH 8.0) was added to the mixture and incubated at 37 °C for 60 min. The reaction was stopped by adding 100 µL of 1 M acetate buffer, pH 4.0. The released p-nitroanilide hydrolysis product was measured at λ = 405 nm. The DPP-IV enzyme inhibitory activity (IC_50_) was expressed as the amount of substance required to half-inhibit the DPP-IV enzyme activity under the conditions described above.

### 2.8. Functional Properties Measurement

#### 2.8.1. Water Absorption Capacity (WAC)

The method was based on Timilsena et al. (2016) [57]. Approximately 1 g of the protein preparation was weighed into a test tube and mixed with 20 mL of distilled water. The mixture was shaken using a laboratory shaker. After 15 min, it was shaken again for 60 s and then centrifuged at 4500× *g* (RCF) for 15 min (Rotofix 32A; Merazet, Poland). Unabsorbed water was carefully decanted, and remaining droplets were removed with blotting paper. The solid phase was dried at 50 °C for 30 min. Water binding capacity (W [g H_2_O/g]) was calculated as:

(3) $$W=\;\frac{C-B}{A}\;$$

where

C—weight of tube with dried sediment (g);

B—weight of empty tube (g);

A—sample weight (g).

#### 2.8.2. Oil Absorption Capacity (OAC)

The procedure followed that of Wu et al. (2009) [58]. One g of protein preparation was mixed with 15 mL of rapeseed oil and shaken. After 30 min, the mixture was at 4000× *g* (RCF) for 10 min. The oil binding capacity (X) was calculated as:

(4) $$X=\frac{V-L}{a}$$

where

V—volume of oil used (mL);

L—volume of unabsorbed oil (mL);

a—weight of the sample, calculated per 100 g of dry matter.

#### 2.8.3. Nitrogen Solubility Index (NSI)

According to Achouri et al. (2012) [59], 200 mg of protein preparation was mixed with 15 mL of distilled water. The pH was adjusted to 2, 4, 6, 8, 10, or 12 using 0.5 M NaOH or 0.5 M HCl. Samples were shaken at room temperature for 30 min, then centrifuged at 4500× *g* (RCF, ≈10,000 rpm) for 15 min (MPW-351 R centrifuge, MPW, Warsaw, Poland, 10,000 rpm). From the supernatant, 10 g was taken for total nitrogen determination using the Kjeldahl method. The total protein content was calculated using a nitrogen-to-protein conversion factor of 6.25, in triplicate. Protein solubility (PS) was calculated as:

(5) $$PS=\frac{A}{B}\;x\;100\%$$

where

A—protein content in supernatant (g);

B—protein content in 200 mg of preparation.

#### 2.8.4. Emulsion Stability

Hydrolysates at 1%, 2%, and 3% concentrations were mixed with 10 mL of water and homogenized until uniform. Pre-emulsification was performed using a lab homogenizer for 2 min at 16,000 rpm (T10 basic ULTRA-TURRAX, IKA WerkeGmbH&Co., Baden-Wurttemberg, Germany). Subsequently, the homogenizer was operated for 20 s, during which a quantity of 10 mL of soybean oil was added. Homogenization continued until 2 min had passed from the first drop of oil (total time: 180 s). The emulsion was transferred to a 10 mL polypropylene tube (14 mm internal diameter). Phase separation (sedimentation) was observed at 30 min, 1 h, 2 h, and 3 h. Emulsion stability (SE) was calculated as:

(6) $$SE=\frac{a}{b}\;x\;100$$

where

a—height of emulsified layer [cm];

b—total emulsion height [cm].

#### 2.8.5. Foam Stability

Into a 1 L beaker, 100 mL of fresh egg white was mixed with hydrolysate (1%, 2%, 3%). The mixture was whipped using a kitchen mixer at 800 rpm for 10 min. The liquid at the bottom was poured into a graduated cylinder. The beaker with foam was left at ~20 °C for 30 min. After 30 min, drainage liquid from the foam was collected and combined with the initial bottom phase for total drainage volume measurement. Foam stability was expressed in millilitres as the total drainage volume and as a percentage [%] of retained foam volume. The control sample contained only fresh egg white, without the addition of any test substances.

### 2.9. Protein Extrudates

#### 2.9.1. Production

The protein isolates and their hydrolysates obtained from *Yarrowia lipolytica* JII1c biomass were processed using a laboratory twin-screw extruder (AEV 650, Brabender). The extrusion was conducted according to the methodology of Rytel, under controlled conditions, with a screw compression ratio of 4:1, a screw speed of 180 revolutions per minute, and a load range of 4.5 to 7 amperes [60]. A die with a diameter of 4 mm was used for shaping the extrudates. To assess the impact of processing temperature, the extrusion was performed at three different temperatures: 160 °C, 170 °C, and 180 °C. These parameters were selected to simulate industrial processing conditions for the development of high-protein, plant-based meat analogues.

#### 2.9.2. Sensory Analysis

The sensory evaluation was conducted by a trained panel (n = 8) in accordance with ISO 13299:2016 guidelines [61]. The assessed attributes included taste, odour, texture, saltiness, and appearance. Evaluations were performed under controlled laboratory conditions (20 ± 1 °C, neutral lighting, individual booths). Samples of extrudates were coded with random three-digit numbers and presented in randomized order to avoid positional bias. Panelists rinsed their mouths with water between evaluations. Each attribute was rated on a 5-point hedonic scale, where 1 indicated “very poor” and 5 indicated “excellent.” Results were expressed as mean values. Bioethics approval for the study was granted by the Rector’s Committee for Ethics in Scientific Research, Wroclaw University of Environmental and Life Sciences (Resolution No. N0N00000.020.1.8.4.2024, dated 21 October 2024).

### 2.10. Statistical Analysis

The analysis was performed with the use of the “STATISTICA 13 PL” program by StatSoft Inc., Tulsa, OK, USA. All determinations were performed in triplicate and presented as average value (X) with standard deviation (SD). Assessment of the significant differences between average values in analysed groups was conducted by variance analysis (ANOVA), followed by a Duncan multiple range test. The level of statistical significance was set at *p* < 0.05.

## 3. Results

**Figure 1:**
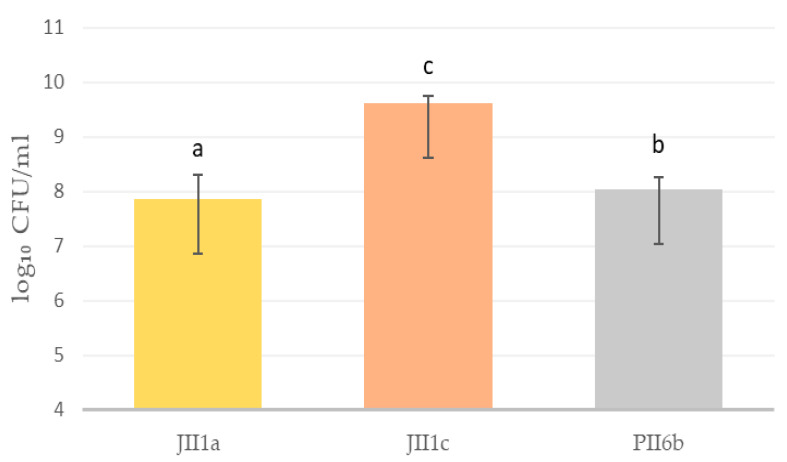
Biomass productivity of selected yeast strains of *Y. lipolytica* in bioreactor cultures [log_10_ CFU/mL]. Small letters (a–c) indicate statistically significant values: a vs. b vs. c (*p* < 0.05), CFU—colony forming units.

A significant productivity difference was observed between the three *Yarrowia lipolytica* strains cultivated under bioreactor conditions (Figure 1). The highest determined cell count was observed in strain JII1c, which reached 9.62 log_10_ CFU/mL, indicating its strong potential for high-yield biomass production. Conversely, strain JII1a exhibited the lowest productivity, measuring 7.86 log_10_ CFU/mL, while PII6b demonstrated an intermediate value of 8.04 log_10_ CFU/mL (1II1a vs. JII1c, *p* = 0.00011; 1II1a vs. PII6b, *p* = 0.04193; JII1c vs. PII6b, *p* = 0.00023). The observed differences in cell density were supported by standard deviation bars, suggesting biological variation across replicates. The markedly higher cell count of JII1c reinforces its selection for subsequent protein isolation, as it combines both high biomass accumulation and favourable compositional traits.

### 3.1. Biomass Yield and Nutritional Composition

The dried biomass of *Yarrowia lipolytica* JII1c appeared as a light beige, free-flowing powder with a characteristic yeast-like odour. Microbiological analysis confirmed its safety, showing <10 CFU/mL viable cells, ≤5 × 10^3^ CFU/mL total aerobic microorganisms, ≤10^2^ CFU/mL yeasts and moulds, and the absence of coliform bacteria and *Salmonella* spp. in 25 g of the sample.

The Nutritional characteristic of dried biomass from *Yarrowia lipolytica* JII1c strain was presented in Table 1. The chemical composition revealed a high dry matter content (95.11%) and a substantial protein level (43.12%), thus confirming the strain’s potential as a protein-rich ingredient. The carbohydrate content constituted 32.34% of the total composition, with the predominant proportion being dietary fibre (32.32%), and negligible levels of sugars (<0.20%), thereby substantiating its compatibility for low glycaemic formulations. The fat content was moderate (7.03%) and primarily composed of monounsaturated (4.05%) and polyunsaturated (3.30%) fatty acids, with saturated fats present at minimal levels (0.50%). The ash content (11.00%) indicated an elevated mineral load, while the low moisture content (4.89%) ensured the desired shelf stability. The salt content was found to be 4.62 g/100 g.

The biomass also proved to be a source of micronutrients relevant to human nutrition. It contained a complex of B-group vitamins, including riboflavin, biotin, folic acid, and vitamin B12—compounds of particular importance in plant-based diets. The mineral profile included considerable amounts of phosphorus, potassium, and sodium, alongside nutritionally relevant levels of iron, zinc, magnesium, and other trace elements.

The amino acid profile indicated the presence of all essential and non-essential amino acids, with lysine (31.1 g/kg), leucine (31.2 g/kg), and valine (25.1 g/kg). The content of methionine + cysteine (9.3 g/kg), tryptophan (5.1 g/kg), and histidine (8.7 g/kg) further emphasized the high nutritional quality of the protein. Furthermore, the presence of gamma-aminobutyric acid (11.0 g/kg) may contribute to functional properties. The fatty acid composition was dominated by oleic acid (48.20%) and linoleic acid (29.20%), with minor contributions from saturated fatty acids such as palmitic and stearic acid. Detailed comparative data on the chemical composition and amino acid profile of the three *Y. lipolytica* strains (JII1a, PII6b and JII1c) are provided in the Supplementary Materials (Table S1).

The protein quality indices indicated that the biomass exhibited high nutritional adequacy, with a chemical score (CS) of 37.80% and an essential amino acid index (EAAI) of 36.17%, calculated relative to whole egg protein. A thorough microbiological analysis was conducted to ascertain the safety of the product. The analysis yielded low levels of aerobic microorganisms, negligible yeast and mould counts, and an absence of coliform bacteria and *Salmonella* spp.

### 3.2. Protein Hydrolysis and Peptide Characterization

**Figure 2:**
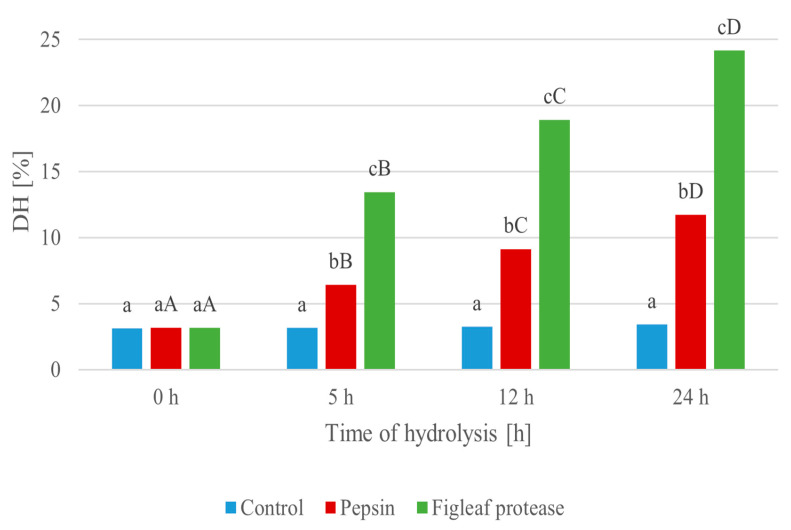
Degree of hydrolysis (DH [%]) during enzymatic degradation of protein extracted from *Y. lipolytica* JII1c yeast biomass, carried out using pepsin and serine protease from figleaf pumpkin at different times of reaction. Small letters (a–c) indicate statistically significant differences (*p* < 0.05) between sampling times within the same enzyme treatment. Capital letters (A–D) indicate statistically significant differences (*p* < 0.05) between enzyme types (control, pepsin, figleaf protease) at the same time point.

The progression of protein hydrolysis (Figure 2) was evaluated over a 24 h period using two enzymatic treatments: pepsin and a serine protease derived from figleaf pumpkin (*Cucurbita ficifolia*). As shown in Figure 2, minimal changes in DH values were observed in the control sample, ranging from 3.12% at 0 h to 3.41% at 24 h, indicating spontaneous, non-enzymatic breakdown of peptides [62]. A higher DH indicates more extensive protein breakdown, which can correlate with increased bioactivity and improved techno-functional properties of the hydrolysates.

The hydrolysis level statistically increased after enzymatic treatment with pepsin, reaching 11.72% after 24 h, with a clear stepwise progression at 5 h (6.42%) and 12 h (9.13%) (0 h vs. 24 h, *p* = 0.000077). Moreover, it is notable that the hydrolysis catalyzed by the figleaf protease yielded a statistically higher degree of hydrolysis (DH) over the course of the experiment, increasing from 3.16% at 0 h to 24.15% at 24 h (0 h vs. 24 h, *p* = 0.000077). The most pronounced increase occurred within the first 5 h (13.45%), indicating strong and rapid proteolytic activity.

Hydrolysate produced using figleaf protease exhibited the highest degree of hydrolysis, confirming the superior efficiency of this plant-derived enzyme in degrading *Y. lipolytica* protein. Consequently, this hydrolysate was selected for further investigation, including the assessment of its biological activities and techno-functional properties. The 24 h hydrolysate was selected for further analysis, including assessment of its biological activities and techno-functional properties.

**Figure 3:**
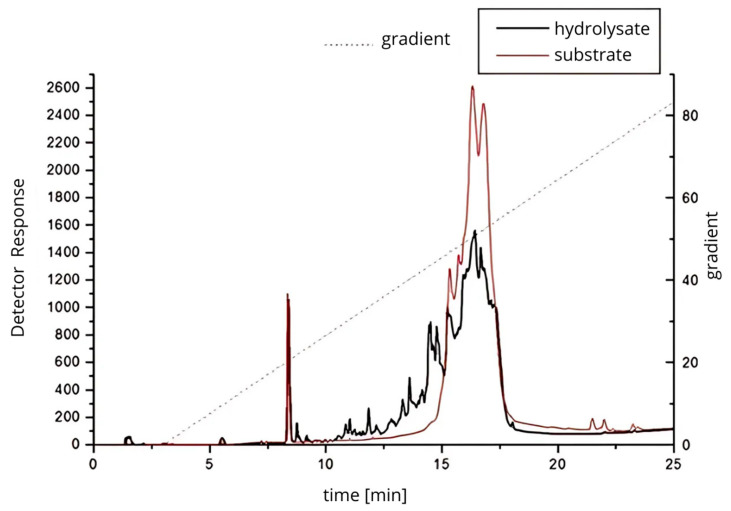
Protein–peptide separation of 24 h protein hydrolysate from *Y. lipolytica* JII1c yeast biomass and digested with serine protease from figleaf pumpkin.

RP-HPLC analysis (Figure 3) demonstrated distinct differences in peptide profiles between the untreated protein isolate and the 24 h hydrolysate generated with *Cucurbita ficifolia* serine protease. The hydrolysate exhibited a broader distribution of peaks and increased peak complexity, particularly between 10 and 20 min of retention time, reflecting extensive proteolysis and the release of low molecular weight peptides. These chromatographic results are indicative of a more heterogeneous mixture of peptides, likely with varying molecular weights and bioactivities, and confirm the potent proteolytic activity of the figleaf pumpkin enzyme, capable of producing diverse peptide fractions.

### 3.3. Biological Activities of Hydrolysates

The 24 h enzymatic hydrolysate of *Yarrowia lipolytica* JII1c protein exhibited significantly enhanced biological activity in comparison with the native protein isolate (Table 2). The antioxidant activity, measured by DPPH and FRAP assays, increased more than twofold (from 0.390 to 0.87 µM Trolox/mg; *p* = 0.000291) and nearly tenfold (from 4.03 to 39.12 µg Fe^3+^/mg; *p* = 0.000191), respectively. Concurrently, the Fe^2+^ ion chelating capacity exhibited a marked enhancement, rising from 275 to 1326 µg Fe^2+^/mg (*p* = 0.000272).

In terms of enzyme inhibition, the hydrolysate demonstrated a substantial decrease in IC_50_ values, indicating enhanced inhibitory potency. ACE inhibitory activity improved nearly fivefold (from 37.43 to 8.20 mg/mL; *p* = 0.000291). Additionally, α-glucosidase and DPP-IV inhibitory activities increased approximately three- to fourfold, with IC_50_ values dropping from 12.23 to 4.17 mg/mL (*p* = 0.000261) and from 10.20 to 2.40 mg/mL (*p* = 0.000181), respectively.

### 3.4. Functional Properties of Protein Preparations

The water absorption capacity of the hydrolysate more than doubled compared to the native protein (from 4.34 g H_2_O/g to 9.12 g H_2_O/g; *p* = 0.000291) (Table 3). This indicates an improved exposure of hydrophilic groups and a higher degree of molecular unfolding after hydrolysis. This facilitates water binding, which is advantageous for plant-based formulations requiring moisture retention.

A similar trend was observed in the OAC, which increased from 2.35 g oil/g in the protein isolate to 7.23 g oil/g in the hydrolysate (*p* = 0.000391). The elevated OAC may be attributed to the generation of smaller peptides with exposed nonpolar residues that interact more effectively with lipids, thereby enhancing the emulsion or mouthfeel properties in fat-containing food matrices.

The analysis of the NSI, which reflects protein dispersibility and processing potential, revealed more than a twofold increase in the hydrolysate compared to the isolate (49.20% vs. 19.40%; *p* = 0.000291).

This enhancement can be attributed to the breakdown of high-molecular-weight proteins into smaller, more soluble peptide fragments, thereby improving the protein’s suitability for use in liquid or semi-liquid food systems.

The stability of egg white foam was found to be significantly influenced by the addition of yeast protein preparations from *Yarrowia lipolytica* JII1c biomass (Table 4). The incorporation of the protein isolate resulted in a notable enhancement of foam persistence, which was observed to be dose-dependent. At the highest concentration of yeast PI (3%), the leakage volume was reduced to 0 mL (100% of retained foam volume), indicating full foam stability, in contrast to 29.0 mL in the control sample without any additive (*p* = 0.000059). Lower doses of yeast protein isolate (1% and 2%) also demonstrated a statistically significant improvement in foam stability, in comparison to the control sample, with leakage reduced to 6.6 mL (77.2% of retained foam volume) and 1.9 mL (93.5% of retained foam volume), respectively.

In contrast, the 24 h hydrolysate from *Y. lipolytica* JII1c showed much lower effectiveness in stabilising the foam (Table 4). Even at the highest tested concentration of 3%, the leakage volume was 2.4 mL (91.7% of retained foam volume), while at 1% and 2% doses, the leakage remained relatively high at 28.0 mL (3.5% of retained foam volume) and 6.8 mL (76.6% of retained foam volume) (*p* = 0.000152), respectively.

The addition of yeast protein preparations from *Yarrowia lipolytica* JII1c resulted in a significant enhancement of emulsion stability in a model oil-in-water system (1:1 ratio). The control sample exhibited a gradual decline in stability over time, dropping from 64.87% at 0.5 h to 53.36% at 3 h. Both the protein isolate and its 24 h hydrolysate demonstrated markedly higher and more sustained emulsion stability.

For the isolate, emulsion stability improved with increasing dose. At 3%, emulsion stability remained above 95% throughout the 3 h period, reaching 97.41% at 0.5 h and 95.11% at 3 h. A similar trend was observed for the 1% and 2% doses, though with slightly lower stability than the highest dose. A notable observation was the superior performance of the hydrolysate in comparison to the isolate, particularly in the initial stages of the test. All concentrations of hydrolysate tested yielded consistent and high stability values, exceeding 96% throughout the entire testing period. The highest result was noted for the 3% dose at 0.5 h (96.86%) and 3 h (95.75%), indicating superior emulsifying properties.

### 3.5. Sensory Evaluation of Extrudates

**Figure 4:**
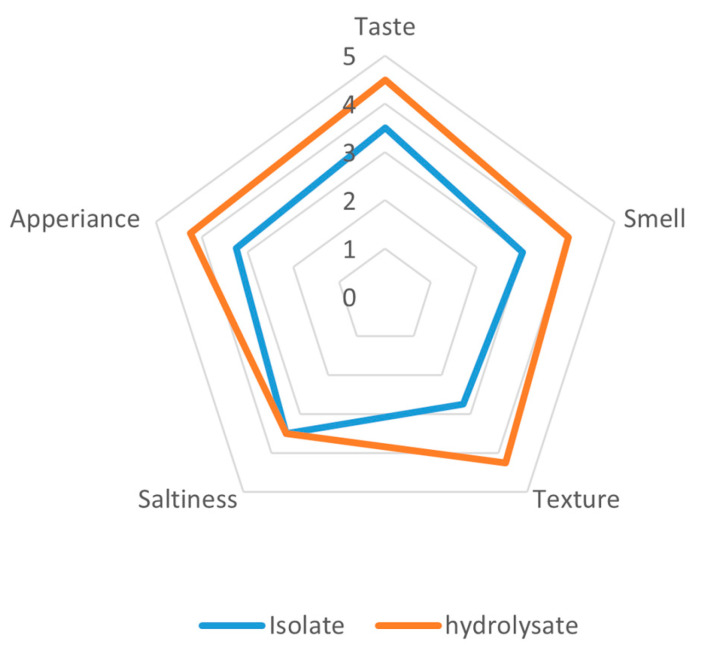
Organoleptic evaluation of extrudates prepared from a protein isolate extracted from *Y. lipolytica* JII1c yeast biomass and its hydrolysate.

The organoleptic analysis of extrudates supplemented with yeast protein preparations from *Yarrowia lipolytica* JII1c revealed a clear preference for the hydrolysate over the isolate across most sensory attributes (Figure 4). The hydrolysate-enriched extrudates obtained higher scores in terms of taste (4.5 and 3.5), smell (4.0 and 3.0), texture (4.25 and 2.75), and appearance (4.25 and 3.25). Notably, saltiness perception was consistently unaltered for both samples at a rating of 3.5, indicating that the process of enzymatic hydrolysis did not affect the perception of saltiness.

The most significant differences were observed in texture and aroma, suggesting that the hydrolysate contributed to more appealing sensory properties, such as mouthfeel and aroma profile, possibly due to the formation of flavour-active peptides during hydrolysis. These findings underscore the potential of protein hydrolysates from *Y. lipolytica* to enhance the sensory profile of extruded food products.

## 4. Discussion

The primary challenge in the utilisation of yeasts in food applications lies in the efficient release of intracellular components through cell wall disintegration. Another crucial aspect is the conversion of proteins into bioactive peptides while maintaining low processing costs. The findings of this study demonstrate that strain selection is a pivotal factor in determining techno-economic feasibility. The findings of this study demonstrate that strain selection is a pivotal factor influencing process efficiency and the potential applicability of yeast-derived proteins in food production [9,63,64,65,66]. Such gains directly support sustainability goals by reducing substrate input and enabling valorisation of industrial side streams.

The nutritional profile of JII1c, characterized by its high protein content and balanced essential amino acid (EAA) pattern, supports its role as an alternative to conventional proteins. This profile is consistent with the ranges reported for *Y. lipolytica* across various substrates and strains [16,67,68,69]. Although sulphur-containing amino acids continue to be a limiting factor, comprehensive protein quality indices suggest that the EAA composition is appropriate for incorporation into plant-based formulations, particularly due to its ability to complement cereal proteins, as reported by Michalik et al. (2014) [68]. In addition, the presence of dietary fibre and low residual sugars is conducive to a low glycaemic design, while the micronutrient package (iron, zinc, B vitamins including B12) contributes nutritional value relevant to vegan diets and reflects the species’ metabolic flexibility on biofuel-derived streams [25,70]. These characteristics are consistent with the regulatory framework of *Y. lipolytica* biomass as a novel food supplement in the EU, which stipulates explicit utilisation thresholds [12,71].

With regard to the processing strategy, enzymatic hydrolysis remains the most effective method of solubilising yeast matrices and tailoring protein functionality through controlled cleavage, exposure of reactive side chains, and modulation of molecular size without damaging the proteins [72,73]. In the present study, a plant-derived serine protease from *Cucurbita ficifolia* was found to exhibit superior proteolytic efficacy in comparison to pepsin, as evidenced by the distinct peptide distributions observed by RP-HPLC. This finding aligns with the current understanding that the selection of enzymes and the prevailing conditions of a reaction (time, pH, enzyme-to-substrate ratio) regulate the ratio of peptides to free amino acids, thereby influencing the balance between functionality and taste [74,75,76,77]. Elevated levels of hydrolysis can lead to an increase in free amino acids, accompanied by a reduction in longer peptides. Consequently, the optimisation process should be oriented towards application-specific endpoints rather than the pursuit of maximal DH alone [75].

Functionally, hydrolysis enhanced water and oil binding and markedly increased solubility, properties critical for moist textures and dispersibility in beverages or semi-liquid systems. Mechanistically, peptide fragmentation and unfolding expose hydrophilic and hydrophobic groups, thereby enabling improved interactions with both aqueous and lipid phases [78,79,80,81]. The observed trade-off between foaming and emulsion stability, with the former being reduced and the latter enhanced, aligns with interfacial principles. This is evidenced by the rapid migration of smaller peptides, which can form cohesive interfacial films that stabilise oil-in-water emulsions. A similar phenomenon has been reported for yeast-derived bioemulsifiers [81].

Beyond functionality, the bioactivities of the hydrolysate were substantially higher than those of the native isolate. This is in line with literature on yeast-derived antioxidant and enzyme inhibitory peptides relevant to cardiometabolic health [5,82,83,84]. Previous studies have demonstrated that the hydrolysate exhibits immunomodulatory, antimicrobial, and anti-inflammatory properties, indicating potential for broader health benefits [85,86,87]. The sensory improvements observed in extrudates are consistent with the enhanced release of amino nitrogen and small taste-active peptides contributing to umami, mouthfulness, and flavour continuity [88]. This relationship between the biochemical activities and sensory perception suggests that peptides responsible for antioxidant and enzyme inhibitory effects may also modulate taste responses, thereby linking nutritional and sensory functionality. Recent findings further indicate that yeast peptides may enhance saltiness perception through receptor interactions, offering a plausible method for reducing sodium levels without compromising flavour intensity [89].

The following section will address the practical implications of the aforementioned points. For manufacturers, *Y. lipolytica* JII1c provides a flexible platform for the generation of application-tuned peptide fractions. The benefits that can be derived from these emulsions and beverages include high solubility and emulsion stability. Meat analogues and baked systems, meanwhile, leverage water/oil binding. Finally, savoury snacks and extruded matrices gain from umami-active peptides and potential salt reduction. The utilisation of a plant protease has been demonstrated to enhance consumer acceptance of processing aids, thereby aligning with prevailing sustainability narratives.

Although the current results highlight the potential of *Y. lipolytica* proteins, there are still several challenges to overcome. The bioavailability and stability of the proteins during processing and storage, as well as their sensory thresholds in complex foods, require confirmation in both model and real systems. Future work should also address bitterness at higher degrees of hydrolysis and identify bioactive peptides. Finally, techno-economic analyses are required to benchmark enzyme costs and scaling-up scenarios.

## 5. Conclusions

*Yarrowia lipolytica* JII1c exhibited elevated levels of protein and significant biomass yield, in addition to high levels of dietary fibre and essential micronutrients (e.g., iron, zinc, B vitamins), indicating its potential as an alternative protein source.

The amino acid profile of the biomass demonstrated a balanced EAA composition, and although sulphur-containing amino acids remained limiting, the overall protein quality (CS and EAAI) supports its use in plant-based nutrition systems.

Enzymatic hydrolysis using serine protease from *Cucurbita ficifolia* markedly enhanced several techno-functional properties of the protein isolates, notably water absorption capacity, oil absorption capacity and nitrogen solubility, which are key attributes for developing novel food formulations. The process also generated bioactive peptides with increased antioxidant and enzyme inhibitory activities that, in addition to their physiological potential, enhanced the perception of umami and saltiness, demonstrating their promise as natural flavour enhancers for reduced-sodium and clean-label food products.

The study highlights the multifunctional character of *Y. lipolytica*, combining nutritional richness, functional versatility, and sensory benefits, which strongly supports its use as a natural, health-promoting ingredient in sustainable food production. The yeast’s natural origin, efficient bioprocess scalability, and ability to valorise agro-industrial by-products further strengthen its relevance to the circular bioeconomy model and to the growing demand for environmentally responsible protein sources.

In order to apply these results in a practical context, it is essential that future research focus on evaluating the bioavailability of the released peptides, their stability during food processing and storage, and comprehensive toxicological and safety assessments for human consumption. Furthermore, the investigation of sensory optimisation, digestive stability, and techno-economic feasibility under industrial conditions will provide a clearer path towards the commercial utilisation of *Y. lipolytica*-derived proteins and peptides in modern nutrition.

## Figures and Tables

**Table 1:** Nutritional composition characteristic of dried biomass from *Yarrowia lipolytica* JII1c strain.

Nutritional Analysis		
Nutrient	Unit	Value [Mean ± SD]
Energy value	kJ (kcal)/100 g	1588 (375)
Dry matter	%	95.11 ± 0.95
Protein	%	43.12 ± 0.43
Carbohydrates, including:	%	32.34 ± 0.32
dietary fibres	%	32.32 ± 0.32
sugars	%	<0.20
Ash	%	11.00 ± 0.11
Fats, including:	%	7.03 ± 0.07
saturated fatty acids	%	0.50 ± 0.01
monounsaturated fatty acids	%	4.05 ± 0.04
polyunsaturated fatty acids	%	3.30 ± 0.03
Water content (moisture)	%	4.89 ± 0.05
Salt content	g/100 g	4.62 ± 0.05
Vitamins	Unit	Value [mean ± SD]
Vitamin B1 (thiamine)	mg/100 g	0.01 ± 0.01
Vitamin B2 (riboflavin)	mg/100 g	3.19 ± 0.14
Vitamin B6 (pyridoxine)	mg/100 g	0.20 ± 0.01
Vitamin B7 (biotin)	µg/100 g	205.00 ± 3.34
Vitamin B9 (folic acid)	µg/100 g	177.00 ± 7.06
Vitamin B12 (cyanocobalamin)	µg/100 g	0.33 ± 0.02
Macro and Microelements	Unit	Value [mean ± SD]
Calcium (Ca)	mg/100 g	27.50 ± 1.49
Phosphorus (P)	mg/100 g	2640.00 ± 13.94
Sodium (Na)	mg/100 g	1848 ± 76.57
Iron (Fe)	mg/100 g	24.00 ± 2.30
Copper (Cu)	mg/100 g	1.90 ± 0.08
Magnesium (Mg)	mg/100 g	138.00 ± 6.12
Potassium (K)	mg/100 g	2665 ± 15.33
Manganese (Mn)	mg/100 g	1.06 ± 0.04
Zinc (Zn)	mg/100 g	22.00 ± 0.77
Chromium (Cr)	mg/100 g	0.10 ± 0.01
Amino Acid Profile	Unit	Value [mean ± SD]
Aspartic acid	g/kg	41.10 ± 0.41
Glutamic acid	g/kg	56.00 ± 0.56
Arginine	g/kg	21.40 ± 0.21
Serine	g/kg	27.70 ± 0.28
Alanine	g/kg	31.10 ± 0.31
Glycine	g/kg	17.20 ± 0.17
Proline	g/kg	16.80 ± 0.17
Lysine	g/kg	31.10 ± 0.31
Methionine + Cystine	g/kg	9.30 ± 0.05
Phenylalanine + Tyrosine	g/kg	35.20 ± 0.35
Threonine	g/kg	25.40 ± 0.25
Tryptophan	g/kg	5.10 ± 0.05
Leucine	g/kg	31.20 ± 0.31
Isoleucine	g/kg	23.60 ± 0.24
Valine	g/kg	25.10 ± 0.25
Histidine	g/kg	8.70 ± 0.09
Ornithine	g/kg	1.10 ± 0.03
Gamma-aminobutyric acid	g/kg	11.00 ± 0.61
Taurine	g/kg	< 0.05
Fatty Acids	Unit	Value [mean ± SD]
Butyric acid (C4:0)	%	<0.05
Myristic acid (C14:0)	%	<0.05
Pentadecanoic acid (C15:0)	%	0.48 ± 0.02
Palmitic acid (C16:0)	%	6.12 ± 0.20
Palmitoleic acid (C16:1)	%	0.38 ± 0.02
Heptadecanoic acid (C17:0)	%	0.19 ± 0.01
Stearic acid (C18:0)	%	1.05 ± 0.04
Oleic acid (C18:1, n9c)	%	48.20 ± 0.87
Linoelaidic acid (C18:2, n6t)	%	<0.05
Linoleic acid (C18:2, n6c)	%	29.20 ± 1.11
Arachidic acid (C20:0)	%	0.19 ± 0.01
cis-11.14-Eicosadienoic acid (C20:2)	%	<0.05
Behenic acid (C22:0)	%	<0.05
cis-11,17,17-Eicosatrienoic acid (C20:3, n3)	%	<0.05
Lignoceric acid (C24:0)	%	<0.05
Nutritional Indexes		
Parameter	Unit	Value [mean ± SD]
CS	%	37.80 ± 0.38
EAAI	%	36.17 ± 0.36

SD—standard deviation; CS—chemical score [%]; EAAI—essential amino acid index [%].

**Table 2:** Level of biological activity in a 24 h hydrolysate solution of protein extracted from *Y. lipolytica* JII1c yeast biomass digested with serine protease from figleaf pumpkin.

Type of Biological Activity Determination Method	Sample	
	PI from *Y. lipolytica* JII1c Biomass	PH from *Y. lipolytica* JII1c Biomass
	Mean ± SD	Mean ± SD
Antioxidant activity (DPPH) [μM Trolox/mg]	0.39 ± 0.01 ^a^	0.87 ± 0.00 ^b^
Fe^3+^ ion reducing capacity (FRAP) [g Fe^3+^/mg]	4.03 ± 0.01 ^a^	39.12 ± 0.01 ^b^
Fe^2+^ ion chelation capacity [μg Fe^2+^/mg]	275.00 ± 0.58 ^a^	1326.00 ± 0.58 ^b^
Angiotensin-converting enzyme (ACE) inhibitory activity IC_50_ [mg/mL]	37.43 ± 0.06 ^a^	8.20 ± 0.00 ^b^
α-Glucosidase inhibitory activity IC_50_ [mg/mL]	12.23 ± 0.12 ^a^	4.17 ± 0.06 ^b^
DPP-IV inhibitory activity IC_50_ [mg/mL]	10.20 ± 0.00 ^a^	2.40 ± 0.10 ^b^

PI—protein isolate; PH—24 h protein hydrolysate; mean values (PI vs. PH) with different letters (a, b) indicate statistically different values (*p* < 0.05).

**Table 3:** Functional properties of the protein isolate extracted from *Y. lipolytica* JII1c yeast biomass and its hydrolysate.

Type of Functional Property	Sample	
	PI from *Y. lipolytica* JII1c Biomass	PH from *Y. Lipolytica* JII1c Biomass
	Mean ± SD	Mean ± SD
Water absorption capacity (WAC) [g H_2_O/g]	4.34 ± 0.04 ^a^	9.12 ± 0.09 ^b^
Oil absorption capacity (OAC) [g oil/g]	2.35 ± 0.02 ^a^	7.23 ± 0.07 ^b^
Nitrogen solubility index (NSI) [%]	19.40 ± 0.19 ^a^	49.20 ± 0.49 ^b^

PI—protein isolate; PH—24 h protein hydrolysate; mean values (PI vs. PH) with different letters (a, b) indicate statistically different values (*p* < 0.05).

**Table 4:** Persistence of egg white foam and emulsion stability of yeast protein isolate *Y. lipolitica* JII1c and its hydrolysate.

Type of Substance	Dose [%]	Leakage Volume After 30 min [mL]	Emulsion Stability [%]			
			0.5 h	1 h	2 h	3 h
		Mean ± SD	Mean ± SD	Mean ± SD	Mean ± SD	Mean ± SD
Control	-	29.0 ± 0.3 ^a^	64.87 ± 0.48 ^a^	56.85 ± 0.48 ^a^	54.07 ± 0.69 ^a^	53.36 ± 0.6
PI from *Y. lipolytica* JII1c biomass	1	6.6 ± 0.1 ^b,A^	90.80 ± 0.72 ^b^	90.49 ± 0.99 ^b^	84.17 ± 0.67 ^b^	83.44 ± 1.32 ^b^
	2	1.9 ± 0.02 ^c,A^	96.80 ± 0.89 ^b^	95.96 ± 1.22 ^b^	95.60 ± 0.53 ^b^	92.92 ± 0.67 ^b^
	3	0.0 ± 0.0 ^d,A^	97.41 ± 1.41 ^b^	96.49 ± 1.43 ^b^	95.74 ± 0.20 ^b^	95.11 ± 0.23 ^b^
PH from *Y. lipolytica* JII1c biomass	1	28.0 ± 0.3 ^a,B^	96.51 ± 1.22 ^b^	96.53 ± 0.59 ^b^	95.88 ± 2.75 ^b^	95.31 ± 2.01 ^b^
	2	6.8 ± 0.1 ^e,B^	96.68 ± 1.04 ^b^	96.47 ± 1.37 ^b^	96.36 ± 0.72 ^b^	95.42 ± 0.54 ^b^
	3	2.4 ± 0.02 ^f,B^	96.86 ± 1.30 ^b^	96.66 ± 1.05 ^b^	96.39 ± 0.41 ^b^	95.75 ± 1.40 ^b^

PI—protein isolate; PH—24 h protein hydrolysate; mean ± SD—average value ± standard deviation; small letters (^a, b, c^ etc.) indicate statistically significant values (*p* < 0.05) for leakage: control vs. PI (1, 2, 3%) and control vs. PH (1, 2, 3%); big letters (^A, B^) indicate statistically significant values (*p* < 0.05) between PI 1% vs. PH 1%, PI 2% vs. PH 2%, and PI 3% vs. PH 3%; small letters (^a, b^) indicate statistically significant values (*p* < 0.05) for emulsion stability: control (0.5, 1, 2, 3 h) vs. PI (1, 2, 3%) and control (0.5, 1, 2, 3 h) vs. PH (1, 2, 3%).

## Data Availability

The original contributions presented in this study are included in the article/Supplementary Materials. Further inquiries can be directed to the corresponding author.

## Supplementary Materials

The following supporting information can be downloaded at: https://www.mdpi.com/article/10.3390/foods14213801/s1, Table S1: Nutritional composition characteristic of dried biomass from *Yarrowia lipolytica* JII1a, JII1c, an PII6b strains.
