# Supplementary material for: Functional, Biological and Nutritional Properties of Protein Fraction Isolated from Yarrowia lipolytica Biomass
Source: Foods. 2025 Nov 6;14(21):3801. doi: 10.3390/foods14213801 (PMC12610664; doi:10.3390/foods14213801)
Supplement: Supplementary file 1 [file foods-14-03801-s001.zip › foods-3931624-supplementary.pdf]

Table S1. Nutritional composition characteristic of dried biomass from *Yarrowia lipolytica* JII1a, JII1c, an PII6b strains

Nutritional Analysis				
**Nutrient**	**Unit**	**JII1a**	**JII1c**	**PII6b**
		**[mean ± SD]**	**[mean ± SD]**	**[mean ± SD]**
Dry matter	%	96,00 ± 0,96[a]	95,11 ± 0,95[a]	94,11 ± 0,94[a]
Protein	%	39,74 ± 0,40[a]	43,12 ± 0,43[b]	38,75 ± 0,39[c]
Carbohydrates, including:	%	34,12 ± 0,34[a]	32,34 ± 0,32[b]	33,25 ± 0,33[c]
- dietary fibers	%	34,08 ± 0,34[a]	32,32 ± 0,32[b]	33,05 ± 0,33[c]
- sugars	%	< 0,40 ± 0,01[a]	< 0,20 ± 0,01[b]	< 0,20 ± 0,01[b]
Ash	%	6,98 ± 0,07[a]	11,00 ± 0,11[b]	6,54 ± 0,07[c]
Fats, including:	%	11,42 ± 0,11[a]	7,03 ± 0,07[b]	12,00 ± 0,12[c]
- saturated fatty acids	%	0,40 ± 0,00[a]	0,50 ± 0,01[b]	0,61 ± 0,01[c]
- monounsaturated fatty acids	%	6,10 ± 0,06[a]	4,05 ± 0,04[b]	5,95 ± 0,06[c]
- polyunsaturated fatty acids	%	4,40 ± 0,04[a]	3,30 ± 0,03[b]	5,38 ± 0,05[c]
Water content (moisture)	%	4,00 ± 0,04[a]	4,89 ± 0,05[b]	5,89 ± 0,06[c]
Salt content	g/100 g	3,74 ± 0,04[a]	4,62 ± 0,05[b]	3,55 ± 0,04[c]
**Amino acids profile**				
Aspartic acid	g/kg	45,2 ± 0,45[a]	41,1 ± 0,41[b]	46,3 ± 0,46[c]
Glutaic acid	g/kg	57,2 ± 0,57[a]	56,0 ± 0,56[b]	54,8 ± 0,55[c]
Arginine	g/kg	20,4 ± 0,20[a]	21,4 ± 0,21[b]	19,7 ± 0,20[c]
Serine	g/kg	28,1 ± 0,28[a]	27,7 ± 0,28[a]	23,5 ± 0,23[b]
Alanine	g/kg	33,3 ± 0,33[a]	31,1 ± 0,31[b]	30,0 ± 0,30[c]
Glycine	g/kg	19,2 ± 0,19[a]	17,2 ± 0,17[b]	20,5 ± 0,21[c]
Proline	g/kg	15,4 ± 0,15[a]	16,8 ± 0,17[b]	14,3 ± 0,14[c]
Lysine	g/kg	29,4 ± 0,29[a]	31,1 ± 0,31[b]	27,1 ± 0,27[c]
Methionine + Cystine	g/kg	6,7 ± 0,07[a]	9,3 ± 0,05[b]	7,0 ± 0,07[c]
Phenylalanine + Tyrosine	g/kg	21,8 ± 0,22[a]	35,2 ± 0,35[b]	26,9 ± 0,27[c]
Threonine	g/kg	23,5 ± 0,23[a]	25,4 ± 0,25[b]	22,7 ± 0,23[c]
Tryptophan	g/kg	4,1 ± 0,04[a]	5,1 ± 0,05[b]	3,80 ± 0,04[c]
Leucine	g/kg	31,0 ± 0,31[a]	31,2 ± 0,31[a]	29,6 ± 0,30[b]
Isoleucine	g/kg	22,5 ± 0,23[a]	23,6 ± 0,24[b]	20,0 ± 0,20[c]
Valine	g/kg	24,5 ± 0,25[a]	25,1 ± 0,25[b]	21,1 ± 0,21[c]
Histidine	g/kg	7,2 ± 0,07[a]	8,7 ± 0,09[b]	6,4 ± 0,06[c]
**Nutritional Indexes**				
CS	%	36,17 ± 0,36[a]	37,80 ± 0,38[b]	31,77 ± 0,32[c]
EAAI	%	30,87 ± 0,31[a]	36,17 ± 0,36[b]	29,54 ± 0,30[c]

small letters (a–c) indicate statistically significant differences ($p < 0.05$) between samples
